# Extreme hypernatremia as a probable cause of fatal arrhythmia: a case report

**DOI:** 10.1186/s13256-016-1062-9

**Published:** 2016-10-01

**Authors:** Maulee Hiromi Arambewela, Noel P. Somasundaram, Chaminda Garusinghe

**Affiliations:** Department of Diabetes and Endocrinology, National Hospital of Sri Lanka, Colombo, Sri Lanka

**Keywords:** Extreme hypernatremia, ECG changes, Hypertonic saline, Elevated intracranial pressure, Case report

## Abstract

**Background:**

Hypernatremia is a frequent occurrence among hospitalized patients. Severe hypernatremia is associated with mortality rates of over 60 %. Extreme hypernatremia, defined as sodium levels >190 mmol/l, is a rare occurrence. The literature on electrocardiographic changes occurring with this degree of hypernatremia is extremely scarce. We report the case of an 11-year-old Sri Lankan girl who presented with sodium levels of 226 mmol/l following infusion with 3 % hypertonic saline who developed diffuse QT prolongation leading to fatal ventricular tachycardia.

**Case presentation:**

An 11-year-old Sri Lankan girl presented with fever, headache, vomiting, and altered level of consciousness. Following admission she developed generalized tonic–clonic seizures and was intubated and ventilated. She had a recent history of polyuria and polydipsia. Magnetic resonance imaging of her brain revealed hydrocephalus due to possible craniopharyngioma. A ventriculoperitoneal shunt was inserted and she was infused with 3 % hypertonic saline in an attempt to reduce intracranial pressure. The following day she became polyuric and dehydrated with tachycardia and low blood pressure. Biochemistry revealed serum sodium of 226 mmol/l, measured serum osmolality of 470 mOsm/kg, urine osmolality of 280 mOsm/kg, urine spot sodium of 116 mmol/l, blood urea of 8.1 mmol/l, and blood glucose of 8.5 mmol/l. Her serum potassium, calcium, and magnesium levels were normal. Extreme hypernatremia due to infusion of 3 % hypertonic saline in the background of cranial diabetes insipidus was considered. She was managed aggressively with 5 % dextrose infusion and clear water via nasogastric feeding to correct the fluid deficit of 7 liters over 36 hours. Her sodium levels dropped to 160 mmol/l the following day. However, she developed electrocardiographic changes with widespread gross QT prolongation with ST segment deviations followed by fatal ventricular tachycardia.

**Conclusions:**

Extreme hypernatremia is rare, and the literature on electrocardiographic changes occurring at such high levels of sodium is scarce. At present there are no established guidelines on rate and mode of correction of such high sodium levels. This case highlights the electrocardiographic changes observed during extreme hypernatremia, controversies in managing increased intracranial pressure with hypertonic saline, and dilemmas encountered in managing extreme hypernatremia.

## Background

Hypernatremia is a frequent electrolyte abnormality observed in hospitalized patients. It is most frequently caused by excess water loss and less frequently by increased sodium intake. Severe hypernatremia (>160 mmol/l) is a serious condition associated with high mortality of >60 % [1]. Cases reported on extreme hypernatremia (>190 mmol/l) are few and literature on electrocardiographic (ECG) changes occurring with this degree of hypernatremia are scarce. Current literature on the management of hypernatremia recommends slow correction in chronic hypernatremia with a 10 to 12 mmol/l reduction in order to prevent adverse neurological sequelae such as cerebral edema [2]. However, there are many controversies in the rate of correction in patients with extreme hypernatremia as persistent hypernatremia is associated with high mortality [3]. Management in such extreme cases poses a therapeutic challenge with some patients achieving successful outcomes with fluid therapy and others with dialysis.

We report the case of a girl with sodium levels of 226 mmol/l following infusion with 3 % hypertonic saline in an attempt to reduce intracranial pressure who developed ECG changes of diffuse QT prolongation and ST segment deviations leading to fatal ventricular tachycardia (VT).

## Case presentation

**Fig. 1:**
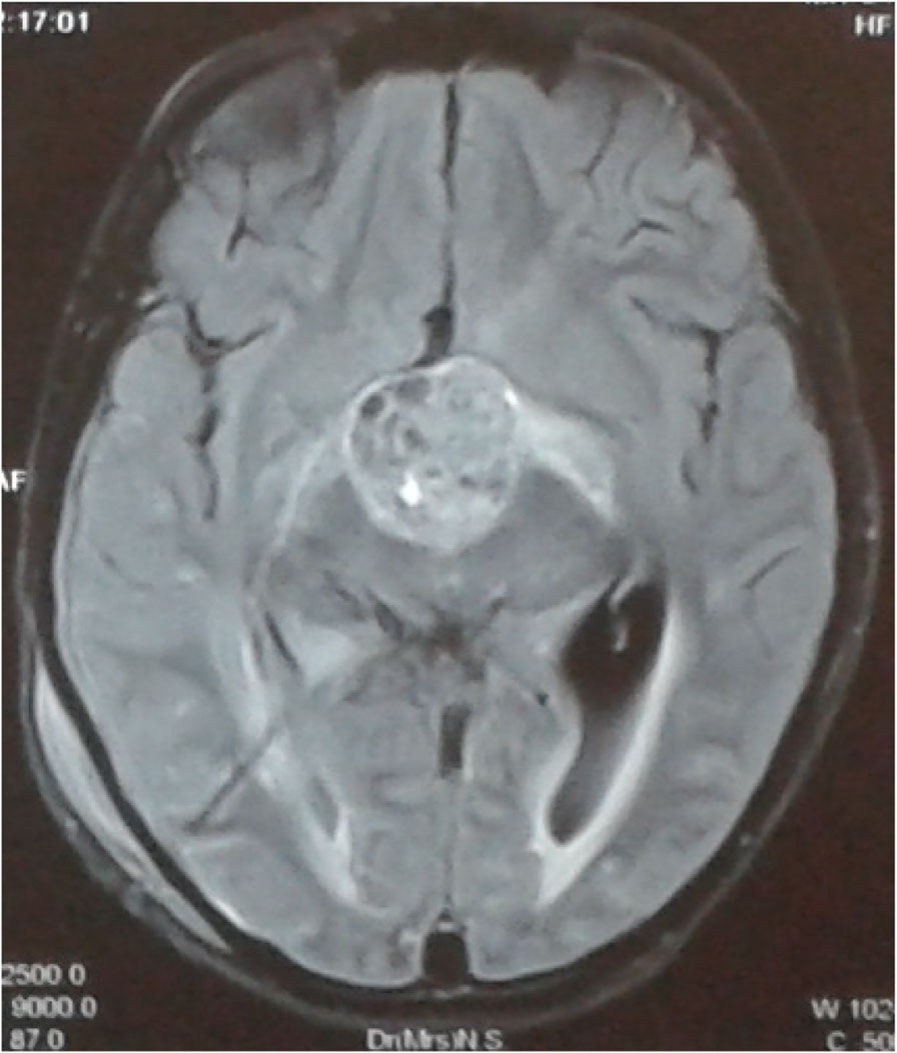
Magnetic resonance imaging T1-weighted pre-contrast axial image showing suprasellar mass suggestive of craniopharyngioma

**Fig. 2:**
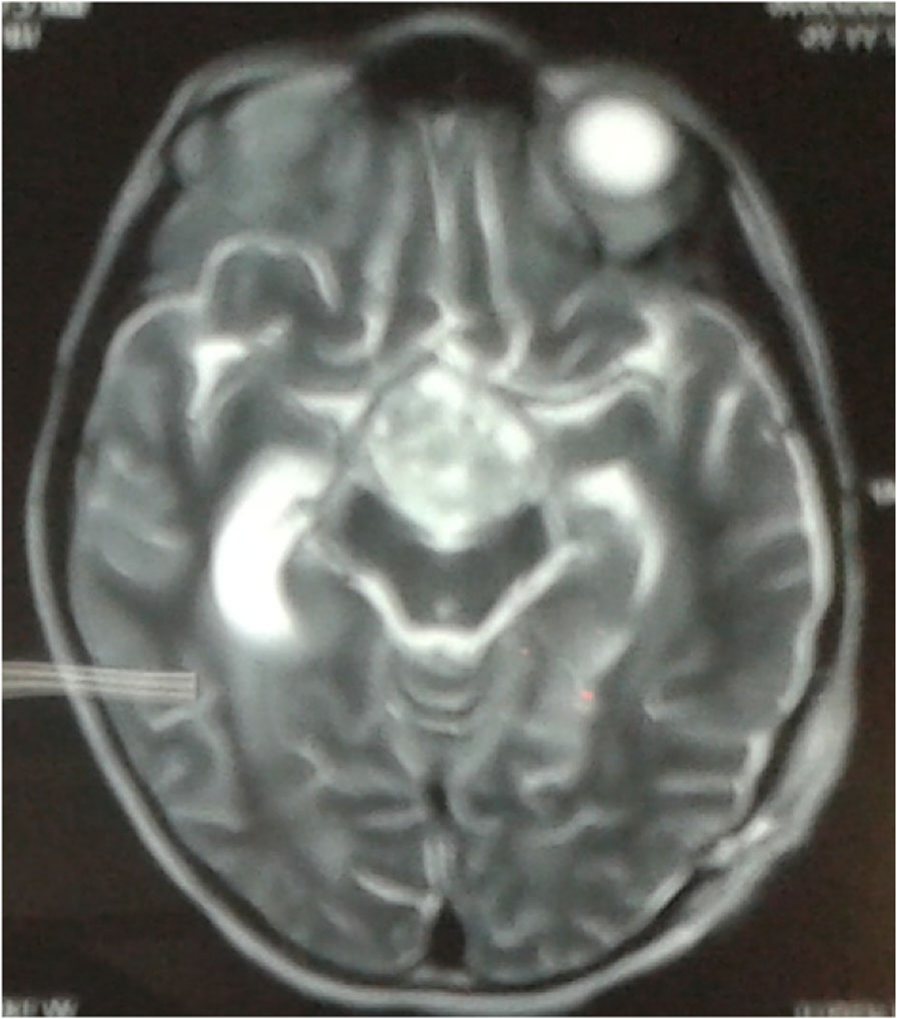
Magnetic resonance imaging T2-weighted pre-contrast axial image of the lesion

**Fig. 3:**
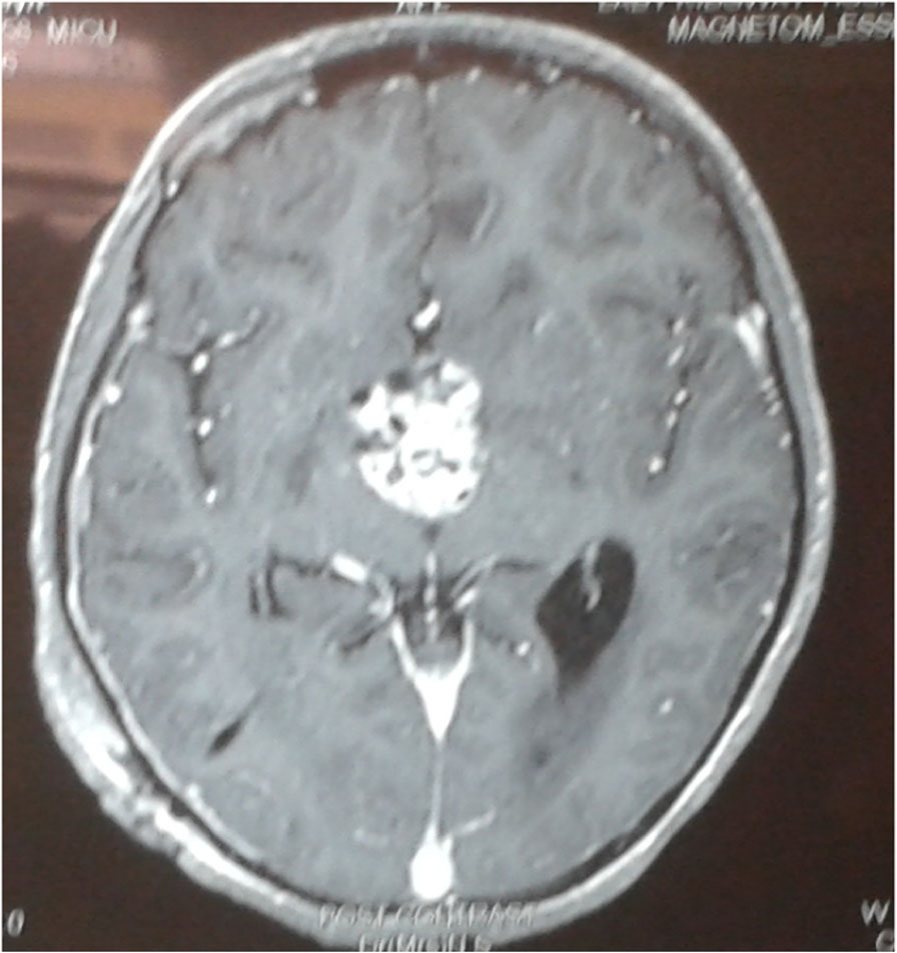
Magnetic resonance imaging post-contrast axial image of the lesion

An 11-year-old Sri Lankan girl presented with a 2-day history of fever, headache, vomiting, and altered level of consciousness. She had been experiencing polyuria and polydipsia during the past 2 weeks. There was no history of chronic headaches, visual disturbances, respiratory symptoms, or abdominal symptoms. She was not known to be diabetic. An examination revealed an unwell-looking febrile girl. Her height was 140 cm (25th centile) and her weight was 25 kg (3rd centile). There was no evidence of neck stiffness or positive Kernig’s sign. Following admission she developed generalized tonic–clonic seizures which required intubation and ventilation. Random blood glucose and electrolyte panel including sodium, potassium, calcium, and magnesium were normal. She was empirically started on antibiotics administered intravenously for possible meningitis. Magnetic resonance imaging revealed gross hydrocephalus with suprasellar mass most likely due to craniopharyngioma (Figs. 1, 2, and 3). A ventriculoperitoneal shunt was inserted and she was started on 3 % hypertonic saline to reduce intracranial pressure. Her basal serum sodium levels were 140 mmol/l and she was commenced on a dose of 5 ml/kg 8 hourly, aiming to increase her sodium levels to 160 to 170 mmol/l. Her serum electrolytes were monitored 8 hourly. Her sodium levels rose to 163 mmol/l after the first 24 hours. It was decided to repeat another dose of 3 % hypertonic saline. However, during the latter part of the day she became polyuric and dehydrated. Her blood pressure was 80/60 and pulse rate was 130 beats per minute. She was hydrated with boluses of normal saline and commenced on hydrocortisone replacement administered intravenously. As her blood pressure failed to stabilize she was started on inotropic support with noradrenaline and dobutamine infusions. Her serum sodium levels were 226 mmol/l and further doses of 3 % hypertonic saline were stopped immediately. Other biochemical investigations were as follows: serum potassium 3.8 mmol/l (3.5 to 5.0 mmol/l), blood urea 8.1 mmol/l (2.5 to 8.0 mmol/l), serum creatinine 76 μmol/l (70 to 120 μmol/l), random blood glucose 8.5 mmol/l, serum ionized calcium 1.12 mmol/l (1.05 to 1.3 mmol/l), and serum magnesium 0.8 mmol/l (0.75 to 0.95 mmol/l). Her measured serum osmolality was 470 mOsm/kg with a urine osmolality of 280 mOsm/kg. There was no discrepancy between measured and calculated serum osmolalities. Her urine spot sodium was elevated at 116 mmol/l. A urine spot sodium of >100 mmol/l can be seen in hypernatremia following ingestion of salt or infusion of 3 % hypertonic saline. Considering the clinical and biochemical factors, hypernatremia in our patient can be attributed to infusion of 3 % hypertonic saline in the background of cranial diabetes insipidus.

**Fig. 4:**
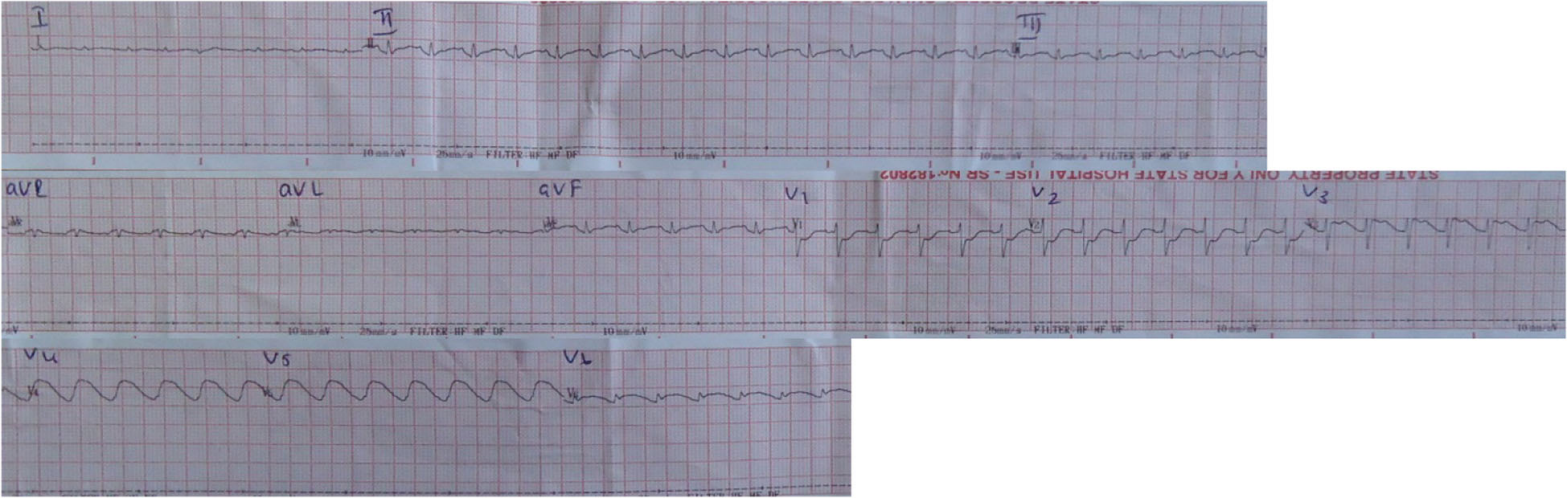
Electrocardiogram showing tachycardia, small complexes, QT prolongation, ST segment deviations, and ventricular tachycardia in V4 and V5

As we considered a sodium level of 226 mmol/l not compatible with life it was decided to correct her sodium levels rapidly. She was treated with an immediate dose of desmopressin 400 micrograms administered orally and hydrated with 5 % dextrose administered intravenously and clear water via nasogastric feeding at a rate of 190 ml/hour to replace the fluid deficit of 7 liters over 36 hours. Her polyuria settled and her serum sodium levels dropped to 160 mmol/l on the following day. However, her cardiac status became unstable and a 12-lead ECG showed diffuse prolongation of QT intervals, ST depressions in leads V1 to V2, ST elevation in V3, and VT in V4 to V5 (Fig. 4). An urgent echocardiogram was planned but she developed VT and went into cardiac arrest. In spite of defibrillation and subsequent resuscitation she died.

## Discussion

Hypernatremia defined as serum sodium levels >145 mmol/l, is associated with increased morbidity and mortality. Severe hypernatremia is defined as sodium levels >160 mmol/l and extreme hypernatremia as >190 mmol/l. Manifestations of hypernatremia vary from nonspecific central nervous system symptoms such as nausea, vomiting, muscle weakness, restlessness, irritability, and lethargy to confusion, nystagmus, seizures, myoclonic jerks, and even death. The level of consciousness is correlated with the severity of hypernatremia. Severe symptoms are likely to occur with acute increases in plasma sodium levels or at concentrations greater than 160 mmol/l [1]. Hypernatremia can cause brain shrinkage, resulting in vascular rupture and intracranial bleeding.

Cases of extreme hypernatremia in humans have been rarely reported and the literature describing ECG changes is scarce. Sodium is the most abundant extracellular cation and its current determines the depolarization and amplitude of the action potential. In Gibson and colleague’s [4] study conducted on the effects of acute alterations in the blood sodium on ECG changes in dogs, rapid induction of hypernatremia showed QT prolongation and decrease in P and QRS amplitude as was seen in our patient. In Gibson and colleague’s study [4], the reduction in the P and QRS amplitude were related to alterations in blood conductivity with a short circuiting effect on myocardial potentials. Prolongation of the QT interval is an indication of an increase in the duration of the action potential due to the high extracellular sodium levels causing an increase in the transmembrane gradient. In a further case of extreme hypernatremia in which a 29-year-old woman had serum sodium of 154 mmol/l, ECG changes of sinus tachycardia, short PR interval, and diffuse ST depressions were observed [5]. A study performed by Fisher *et al*. [6] demonstrated that hypernatremia following acute subarachnoid hemorrhage was associated with adverse cardiac outcomes such as left ventricular contractile dysfunction, elevated cardiac enzymes, pulmonary edema, and death. The actual pathophysiology of hypernatremia on cardiac dysfunction is unknown. It is, however, hypothesized that increased extracellular sodium causes more calcium to exit the cell via sodium calcium exchanger on the sarcolemma. This results in reduced levels of intracellular calcium levels available for cardiac myocyte contraction causing a negative inotropic effect. Considering these facts, extreme hypernatremia would have been the most likely culprit for the fatal arrhythmia that occurred in our patient.

Our patient was given an infusion of 3 % hypertonic saline in an attempt to reduce intracranial pressure. In the last decade many studies have been done on the effects of 3 % hypertonic saline in intracranial pressure in traumatic or non-traumatic brain injury in experimental and human models [7, 8]. Side effects include rebound increased intracranial hypertension, renal impairment, subarachnoid hemorrhage, central pontine myelinolysis, high urinary water losses, and masking of diabetes insipidus. Further studies have shown that the prognosis in children with increased intracranial pressure having higher levels of sodium within the range of 150 to 170 mmol/l and higher levels of serum osmolality of 300 to 340 mOsm/kg appear to be better than normal to low levels of serum soidum and osmolality. However, no patient with a serum sodium >180 mmol/l has had a good outcome [9]. There is Class II evidence supporting the use of hypertonic saline for the acute treatment of severe pediatric traumatic brain injury associated with increased intracranial hypertension and Class III evidence to support its use as a continuous infusion [10]. However, there are insufficient data to determine when to initiate, the duration of treatment, and target sodium concentrations. Further studies are required to resolve these concerns.

Management of such extreme cases of hypernatremia poses a therapeutic challenge. Hypernatremia initially causes fluid to move out of the brain leading to cerebral contraction causing alterations in mental status. To counteract this, the brain initially takes up electrolytes to reduce the contraction of cerebral volume. This is followed by a slower adaptive phase in which there is accumulation of organic osmolytes. Subsequently, the brain volume is restored as the solutes within the brain cells drag water into the cells and thereby restore the cerebral volume within 1 to 3 days [11]. When correcting hypernatremia, awareness regarding these acute and chronic stages is necessary as major changes in osmolality can eventually lead to cerebral edema.

Most literature published on the management of chronic hypernatremia recommends a slow correction rate of 10 to 12 mmol/24 hours [2]. However, no prospective studies validate such recommendations. Alshayeb *et al*. carried out a study on correction rates in severe hypernatremia among hospitalized patients; they concluded that slower correction rates were associated with high mortality [3]. Cases published on extreme hypernatremia are scarce and the rate of correction and modes of correction differ. Most such cases with successful outcomes have achieved more rapid corrections without neurological sequelae [12–16]. A faster correction rate has a risk of causing cerebral edema and a slower rate will promote persistent hypernatremia. Both scenarios are associated with high mortality. However, in the cases with positive outcome, high correction rates were used based on the theory that extreme hypernatremia is not compatible with life. The therapeutic modes used to achieve these higher correction rates differ: fluid therapy, hemodialysis, and peritoneal dialysis.

In our patient, extreme hypernatremia (sodium level of 226 mmol/l) was thought to be acute due to infusion of 3 % hypertonic saline plus underlying cranial diabetes insipidus. Correction was done initially with clear water through nasogastric tube and 5 % dextrose to achieve the fluid deficit of 7 liters during the first 36 hours. Her serum sodium dropped to 160 mmol/l on the following day and fluid replacement was changed to 0.45 % normal saline with clear water at a lower rate. Several cases of extreme hypernatremia have been managed successfully with fluid therapy without any adverse neurological sequelae [12, 15]. However, this was not the case in our patient who succumbed to a fatal arrhythmia. The rate of water replacement in severe hypernatremia is controversial and the available data are insufficient to enable consistent recommendations in such extreme cases.

Even though dialysis is not recommended in present guidelines on hypernatremia, it is believed to have a beneficial role in managing cases of acute extreme hypernatremia. Correction with fluid would require large volumes and longer duration, whereas with dialysis the duration of treatment can be curtailed. Furthermore, dialysis has beneficial effects in scenarios complicated with cardiopulmonary or renal disorders. The rate of correction can be adjusted by changing the concentration of sodium in the dialysate. Few cases of hypernatremia corrected with hemodialysis have been reported without any neurological complications despite the faster correction rate [14, 16]. A safer approach in chronic hypernatremia would be continuous renal replacement therapy where sodium levels can be drawn every hour, and the fluid rate in addition to dialysate concentration can be changed easily [16]. Our patient was initially on inotropic support. However, with fluid resuscitation the inotropes were being gradually withdrawn. Whether she would have benefited from dialysis and, if so, from what form of dialysis, are questions to ponder on.

## Conclusions

Extreme hypernatremia is a rare occurrence and literature on ECG changes in such patients is scarce. Our patient presented with acute extreme sodium levels of 226 mmol/l following infusion with 3 % hypertonic saline in an attempt to reduce intracranial pressure. Even though rapid correction of sodium was done via fluid therapy she developed fatal arrhythmias. ECG revealed gross diffuse QT prolongation with ST depressions and elevations which ultimately led to VT. This case highlights the rare ECG manifestations in extreme hypernatremia, controversies in target sodium levels when attempting to reduce intracranial pressure with hypertonic saline, and dilemmas in managing extreme hypernatremia.

## Abbreviations

ECG: Electrocardiographic
VT: Ventricular tachycardia
